# Reconstruction of the Discourse on Influenza During Pandemics Between 1889 and 1980 in the Predecessor Journal of Swiss Medical Weekly - A Narrative Review and Bibliometric Analysis

**DOI:** 10.3389/phrs.2025.1608522

**Published:** 2025-10-21

**Authors:** Natalija Radivojevic, Milo Puhan, Kaspar Staub, Tala Ballouz

**Affiliations:** 1 Institute of Evolutionary Medicine, Faculty of Medicine, University of Zurich, Zurich, Switzerland; 2 Department of Epidemiology, Epidemiologiy, Biostatistics and Prevention Institute, Faculty of Medicine, University of Zurich, Zurich, Switzerland

**Keywords:** historical trends, historical epidemiology, pandemic, narrative, knowledge

## Abstract

**Objectives:**

Understanding how discourse surrounding influenza pandemics evolves over time can reveal shifts in scientific and public health priorities. This study reconstructs such discourse between 1889 and 1981 by identifying and comparing key research themes and trends in articles in predecessor journals of Swiss Medical Weekly (SMW).

**Methods:**

A corpus of articles containing the terms “influenza,” “flu,” and “epidemic” from five pandemic periods was compiled. We conducted a keyword-based content analysis, categorizing articles into 31 sub-keywords within five broad categories, and comparing their frequencies. Co-occurrence maps were generated using VOSviewer, and relevant articles were closely read.

**Results:**

A total of 352 articles were identified, with the highest number published between 1918 and 1924. Most prevalent themes across all periods were “Epidemiology and disease dynamics,” “Complications,” and “Acute clinical manifestations and management.” A key shift in focus occurred with the introduction of influenza vaccination after the 1940s, as attention increasingly turned towards immunity and the role of vaccinations.

**Conclusion:**

Despite major medical developments, similar themes were seen across the observed pandemic periods in SMW. This study provides an important first step towards constructing such discourses.

## Introduction

During the 19th and 20th century, various epidemics and pandemics significantly impacted public health. Major influenza pandemics in 1889, 1918, 1957, and 1969 led to substantial morbidity and mortality, severely affecting economies and societies worldwide and exposing the vulnerabilities of public health systems [1]. For example, although the 1918-20 influenza pandemic was the greatest demographic catastrophe of the 20th century in Switzerland [2], it created a sense of urgency to understand and combat the disease more effectively, prompting advancements in virology and epidemiology. It also brought attention to long-term neurologic and psychologic sequalae which were frequently encountered after infection [3, 4]. Meanwhile, the 1957 and 1969 pandemics underscored the importance of vaccine development and distribution.

While there are some parallels across these pandemics, each arises and evolves within a specific societal and political context and was influenced by contemporary medical knowledge. This distinctiveness highlights the need to reconstruct discourses within the scientific community during each of the pandemics not only to understand what challenges physicians and scientists encountered at the time but also the ways in which science interacted with society and policy. With such analyses we can see how research focus and public health priorities have shifted over time in response to these crises, while also revealing what debates and uncertainties have persisted. Written media and scientific sources are an essential repository of academic knowledge, providing a comprehensive record of our evolving understanding of diseases and documentation of clinical experiences and outcomes.

The “Correspondenzblatt der Schweizer Ärzte”, what later became today’s Swiss Medical Weekly (SMW), was first published in January 1871. The journal served as a critical platform for the dissemination of medical knowledge and research in Switzerland since then. With its historical roots going back for more than 150 years, the journal has a long history of publishing evidence on various topics including infectious diseases, public health and clinical practice. An examination of the discourse in SMW before and after pandemics provides a valuable resource for analyzing how the Swiss medical community responded to crises and how these responses evolved over time.

Various methods, such as content and bibliometrics analyses [5, 6], can be applied to reconstruct scientific discourse by quantitatively and qualitatively analysing the knowledge landscape and historical trends of a specific research theme or field. They involve systematically examining metadata and texts of journal articles, books, grey literature as well as websites and social media posts, to uncover emerging or recurring themes, patterns and trends over time. These approaches have been applied across multiple research fields including healthcare, sociology, education, and environmental sciences among others [7–15]. In the context of pandemics and epidemics, they can be used to show how research priorities shift in the face of an outbreak, reflect health policy decisions, highlight the development and implementation of a new medical intervention, track the dissemination of information to the public, and reveal the impact of influential works on the scientific community’s understanding of infectious outbreaks [16–23]. For example, for the COVID-19 pandemic, these methods were used to assess research productivity and whether there has been a shift in biomedical scientific production towards COVID-19 publications (“covidisation”) or map the temporal evolution of COVID-19 related themes [22–26].

In this study, we aimed to analyze the nature and content of pandemic related research in Switzerland before, during and after major pandemics between 1889 and 1981. Specifically, we sought to 1) synthesize pandemic research through a review of all published evidence on influenza in the SMW around major pandemic periods, 2) identify key research themes and topics through examining the frequency and co-occurrence of keywords and 3) compare the thematic evolution across different pandemic periods to better understand broader trends in medical research and public health priorities during those times. In the discussion, we also contextualized our findings within the broader context of each of the pandemics, taking into account the literature on public health policies at the time [27–29].

## Methods

### The Selected Pandemic Periods

In this article, we focus on worldwide pandemics of the late 19th and the 20th century that have been proven or are highly likely to have been caused by the influenza virus, because in such events a large proportion of the population falls ill. There is no standardised definition of the pandemic years of these historical pandemics. We have aligned ourselves with the literature and defined the years 1889/90, 1918-20, 1957, 1968-70 and 1977 as pandemic years, and added around 4 years in each case as pandemic periods, on the one hand to take into account the delay in the publication process, and on the other hand to be able to see how publication activity changed in the first years after a pandemic. A summary of selected pandemics from a Swiss perspective as well as a summary of the history of the selected journal can be found in the Supplementary Material.

### Identification and Selection of Articles

We took a two-step approach in which we A) provide a broad overview on journal content over the course of a 100 years and B) look more closely at articles published within selected pandemic periods.

#### Broad Overview 1880 to 1980

All issues of the journal were screened, and the following parameters per year were extracted: number of issues, total number of pages of all issues, and via the detailed subject registers the number of articles on ‘flu’ and ‘influenza’ as well as their total number of pages (Supplementary Table SA3).

#### Selected Pandemic Periods

Because we were particularly interested in the discourse in the context of historical pandemics and the subsequent years after each pandemic ended, we took a closer look at the periods 1889–1894, 1918–1924, 1957–1961, 1968–1974 and 1977–1981. The subject registers of all annual volumes were searched for articles on terms relating to ‘flu’, ‘influenza’, ‘epidemic’ and ‘infectious diseases’ in English (in the latter two criteria, only articles on influenza were subsequently included), German (Influenza, Grippe, Epidemie, Infektionskrankheit), French (Influenza, la grippe, épidémie, maladies infectieuses) and Italian (influenza, epidemia, malattie infettive). This procedure was identical for all selected pandemic periods. In total, we identified and included 352 articles (Supplementary Table S1). The author, issue/publication date, study type or design (i.e., observational, randomized controlled trial, reviews, and opinion articles), article type (i.e., weekly report, original work, meeting/conference reports, cantonal correspondence, surveillance reports, short reports, research summaries of studies published in other journals, and special articles), and length (in pages) were extracted from each article. “Special articles” refers to a series of articles published in 1970 that summarized historical medical milestones and topics which were discussed in SMW over its 100-year history, including a few paragraphs on historical pandemics.

### Assignment of Sub-keywords and Keyword Categories

Each of the selected 352 articles was read and summarized by two study members independently. Based on these summaries, the articles were then generically annotated with 31 different sub-keywords according to their content (and not the interest of the study team) by the two study members (Supplementary Table S1). These sub-keywords were then categorised into five broad keyword categories: “Recovery” (after the acute phase), “Acute clinical manifestations & management”, “Complications” (during the acute phase), “Immunity & vaccination” and “Epidemiology and disease dynamics” (Supplementary Table S2). The “Acute clinical manifestations & management” category includes sub-keywords such as “acute clinical feature,” “secondary infection” and “therapy.” Sub-keywords such as “complication,” “association with Encephalitis lethargica” and “association with psychiatric disorders” fall under the category of “Complications”. The “Epidemiology and disease dynamics” category contains sub-keywords such as “etiology”, “epidemic course”, “prevention” and “histopathological finding.” The “Immunity & vaccination” category includes sub-keywords such as “immunity,” “vaccination” and “reinfection.” The final category, “Recovery,” consists of only one sub-keyword: “recovery course.”

### Methods and Visualisation

To provide an overview of the entire period from 1880 to 1980 (see A above), we first relativised the annual number of articles on influenza and influenza by the number of issues in the respective years, allowing us to account for variations in publication frequency over the years. Second, we relativised the annual number of pages on influenza by the total annual number of all pages. Third, we compared these two-time series visually with the officially reported deaths due to influenza in Switzerland [30, 31] to identify any trends between publication count and length and death rates.

For each pandemic period, we descriptively analysed article numbers, types, and study designs. To detail topic changes (see B above), we compared keyword categories across five pandemic periods using bar and spider charts. Based on main keyword categories, relevant articles were reread and summarised via close reading. We used VOSviewer (v1.6.20), a common bibliometric tool [32–34], to generate keyword co-occurrence maps by clustering sub-keywords. It calculates co-occurrence frequency, cluster strength, and visualises links and total link strength [35]. We selected “Create a term co-occurrence map based on text data” with full counting, minimum occurrence set to 1 (or 3 for 1918 due to higher volume), and default settings except for modified “Attraction” and “Repulsion” parameters. Line thickness indicated connection strength. VOSviewer was not used for the 1968–1974 and 1977–1981 periods due to low article counts. Python (v3.12) and R (v4.4.0) supported additional analyses and visualisations.

## Results

### Broad Overview 1880 to 1980

**FIGURE 1:**
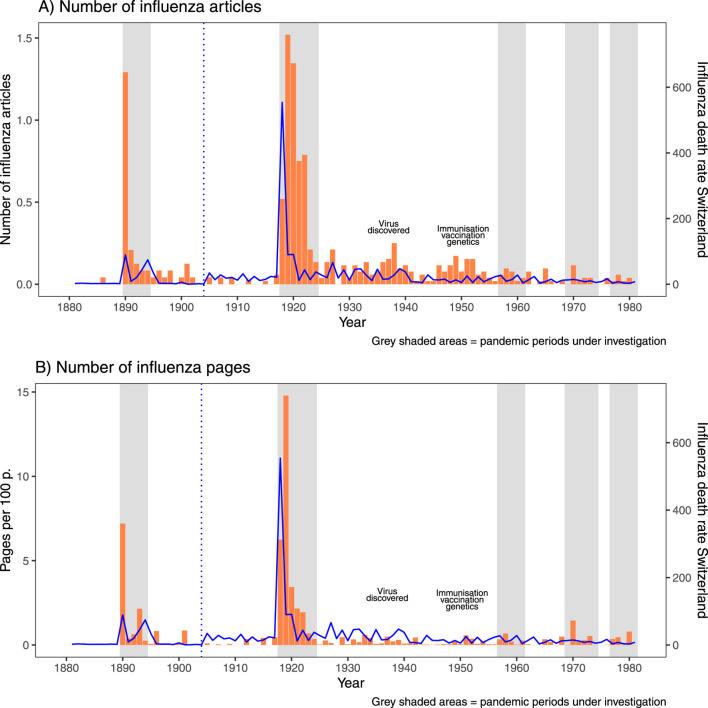
The broad annual overview of influenza mortality in Switzerland compared to the influenza coverage in Swiss Medical Weekly, **(A)** number of articles per issue and **(B)** number of pages per 100 pages. Blue dotted vertical line = change in causes of death reporting of influenza in 1904; grey shaded bars = the pandemic periods examined in depth (Zurich, Switzerland. 2025).

The annual trend in influenza mortality in Switzerland since around 1880 is shown in Figure 1A. Of the official pandemics (1889/90, 1918-1920, 1957, 1968-70 and 1977), only the 1889 and 1918 influenza pandemics led to a clear increase in influenza-related mortality. The subsequent pandemics did not result in higher mortality rates than strong seasonal influenza waves (e.g., 1927, 1929, 1944, etc.). If we compare the temporal synchronicity with the annual number of influenza articles per issue (Figure 1A) and the annual number of pages on influenza per 100 total pages in SMW (Figure 1B), we can see that the two pandemic outbreaks mentioned above, 1889 and 1918, are also clearly reflected in SMW. While the publication peak for the 1890 influenza pandemic already occurred in the first pandemic year 1890 (the peak of the outbreak was in January 1890), the peak for the 1918 pandemic came somewhat later in 1919 and 1920. Notably, the publication coverage was still comparatively high in 1921 and 1922. In terms of influenza articles per issue, the peak in 1890 (1.29 articles per issue) was almost as high as in 1919 and 1920 (1.27 and 1.38 articles, respectively). This is partly because in 1890, SMW still published 24 issues per year, whereas in 1919 it was already 52. Between and after the publication peaks around 1889 and 1918 pandemics, the topic of influenza in SMW almost disappeared repeatedly, with an overall decreasing trend over the 20th century. However, two temporary coverage rebounds can be seen: one around the discovery of the influenza virus in the 1930s and another around 1950 coinciding with increased discussions on viral genetics and vaccination.

### Selected Pandemic Periods

Most articles appeared in the first two pandemic periods 1889–1894 (n = 44) and 1918–1924 (n = 274) (Table 1). In terms of article type, surveillance/weekly reports and cantonal correspondence were still relatively important in the first pandemic period (57%); in the later pandemic periods with the evolution of official public health reporting practices, this article category almost completely disappeared from SMW. In the 1918–1924 pandemic period, research summaries were still relatively important, but in later periods this type of article almost disappeared and mostly original papers on the influenza topic were published. In terms of study design, RCTs only came into play in the last two periods with the advancement of antiviral medications and vaccination, while observational studies and summaries declined over time.

**TABLE 1:** Characterisation of the included articles into article type and study type with indication of the frequency and proportion per pandemic period. The percentages have been rounded mathematically, which may cause them to not total exactly 100% (Zurich, Switzerland. 2025).

	1889–1894, N = 44	1918–1924, N = 274	1957–1961, N = 15	1968–1974, N = 12	1977–1981, N = 7
**Article type**					
Research summaries	0 (0%)	159 (58%)	9 (60%)	2 (17%)	1 (14%)
Original work	6 (14%)	59 (22%)	4 (27%)	6 (50%)	5 (71%)
Meetings/Conference reports	12 (27%)	34 (12%)	1 (7%)	0 (0%)	0 (0%)
Short reports	0 (0%)	22 (8%)	0 (0%)	0 (0%)	0 (0%)
Surveillance reports	13 (30%)	0 (0%)	0 (0%)	0 (0%)	0 (0%)
Weekly report	8 (18%)	0 (0%)	0 (0%)	0 (0%)	0 (0%)
Cantonal correspondance	4 (9%)	0 (0%)	0 (0%)	0 (0%)	0 (0%)
Special article (old papers)	0 (0%)	0 (0%)	0 (0%)	4 (33%)	0 (0%)
Other	1 (2%)	0 (0%)	1 (7%)	0 (0%)	1 (14%)
**Grouped study type**					
Observational	30 (68%)	201 (73%)	13 (87%)	5 (42%)	4 (57%)
Opinions and Summaries	12 (27%)	42 (15%)	0 (0%)	0 (0%)	0 (0%)
Reviews and overviews	2 (5%)	30 (11%)	2 (13%)	5 (42%)	2 (29%)
RCT	0 (0%)	0 (0%)	0 (0%)	2 (17%)	1 (14%)
Corrections	0 (0%)	1 (0.4%)	0 (0%)	0 (0%)	0 (0%)

**FIGURE 2:**
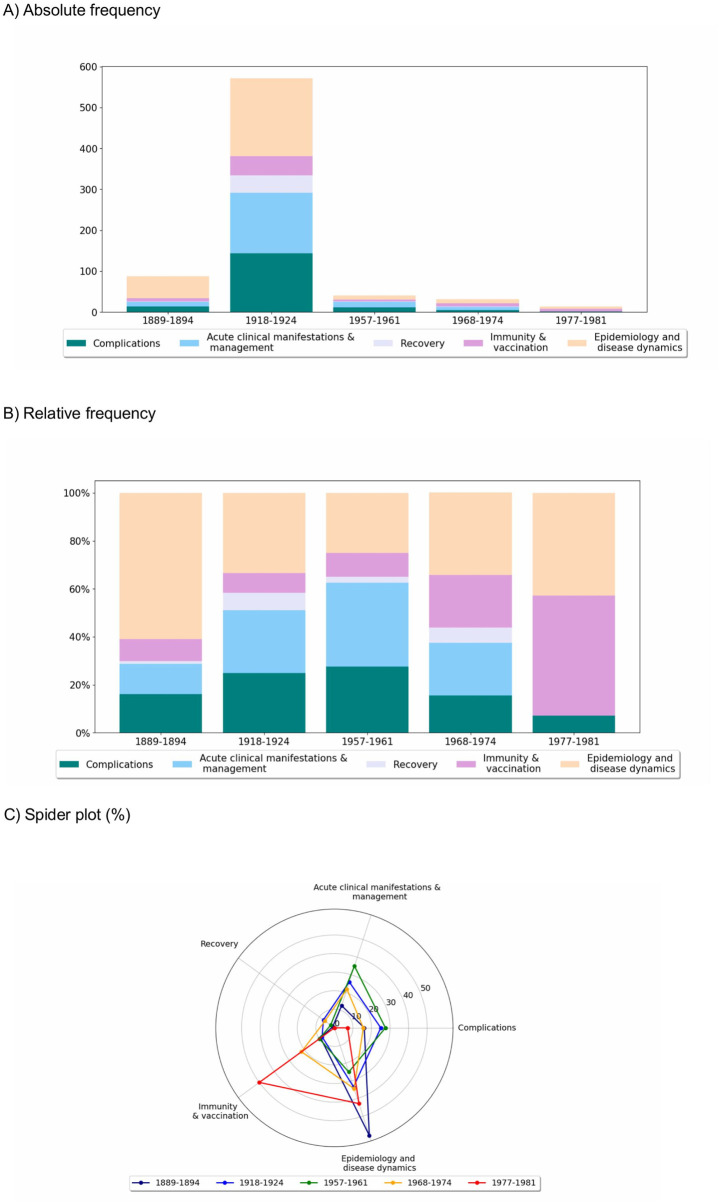
Absolute frequency of occurrences of the five broad keyword categories (panel **(A)**). Relative proportion of the five broad keyword categories within the total mentions for each selected pandemic period as bar plot (panel **(B)**) and spider plot (panel **(C)**), with 100% representing the total mentions for each of the entire pandemic periods (Zurich, Switzerland. 2025).

The absolute frequency of the broad keyword categories is by far the highest in the 1918–1924 pandemic period (a total of 572 mentions, Figure 2A), which also reflects the much higher number of articles in this pandemic period. The second highest frequency of the keyword categories is found in the 1889–1894 pandemic period (87 mentions). The later pandemic periods fade away in comparison. When examining the proportions of the five keyword categories for each selected pandemic period (Figures 2B,C), the “Epidemiology and disease dynamics” category was always relatively important, but most so in the first pandemic period in the context of the 1918 pandemic where the etiology of influenza was still unclear and widely debated. Both the “Complications” (7%–28%) and “Acute clinical manifestations and management” (0%–35%) categories were commonly mentioned across different pandemics, but tended to become less important with time, likely due to improved understanding of the disease, as well as advancements in public health measures and medical practices as the disease became less severe. The category “Recovery” was also mentioned across most of the pandemic years, particularly in the 1918–1922 period, but its share was always rather negligible. Finally, “Immunity and vaccination” became increasingly important in the two most recent periods. Below, we provide a more detailed description of the articles from the five pandemic periods.

### 1889–1894 Pandemic Period

For the pandemic period 1889–1894, a total of 44 papers were identified. The highest number of 31 articles was published in 1890, then rapidly declining to five in 1891, three in 1892 and two in 1893 and 1894. In the first year of the pandemic, 1889, no articles on the subject of influenza were found. This is likely because the pandemic emerged in the winter and researchers needed time to publish their findings. Articles were mainly surveillance reports or meeting/conference summaries (Table 1).

**FIGURE 3:**
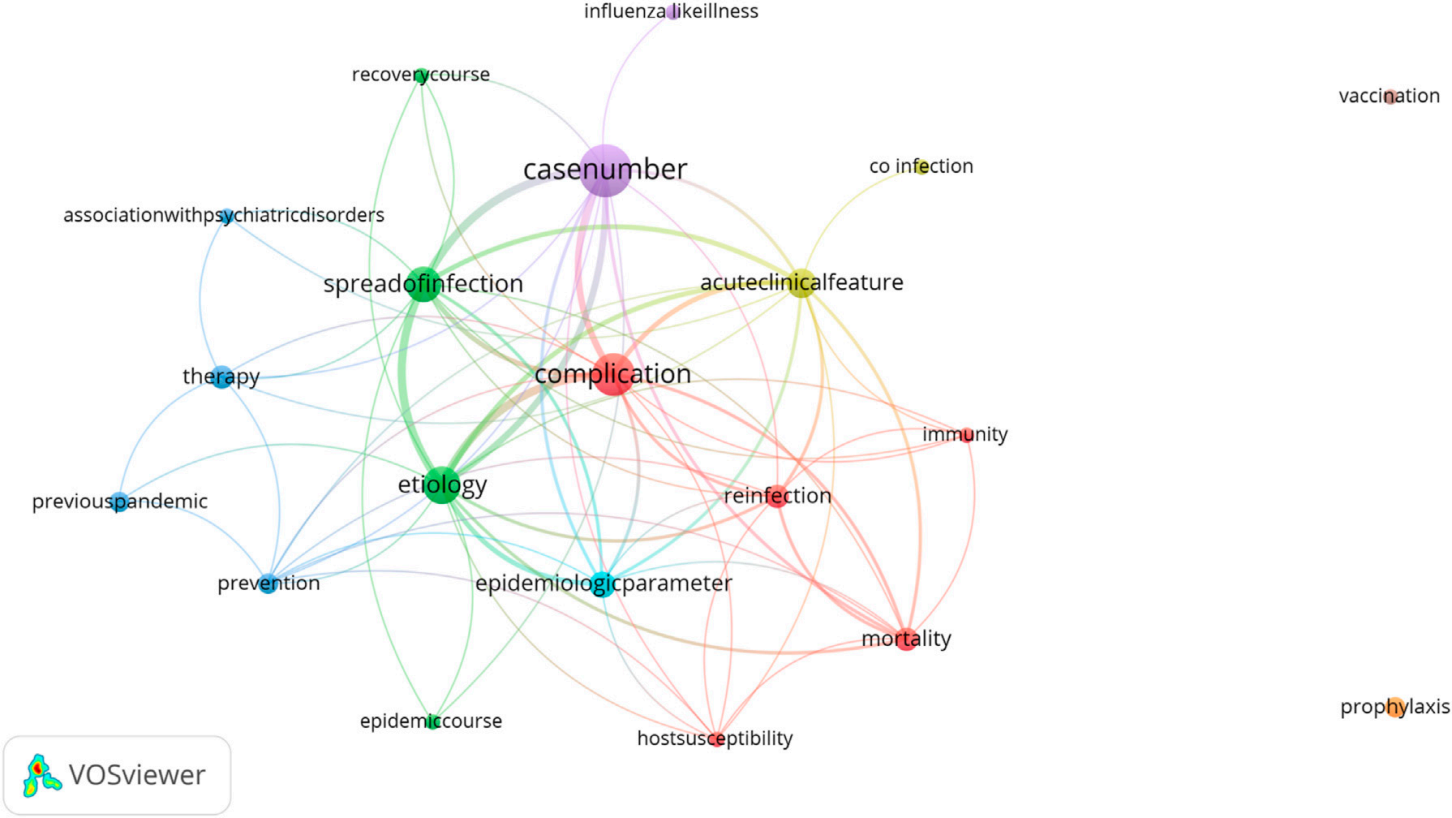
Co-occurrence and frequency map of the sub-keywords for the 1889–1894 pandemic period. The size of the dot indicates the frequency of occurrence of the given sub-keywords. Different colours refer to the different clusters identified by VOSviewer. The thickness of the connecting lines indicates the strength of co-occurrence between sub-keywords (Zurich, Switzerland. 2025).

The VOSviewer cluster analysis of the total of 20 sub-keywords in this first pandemic period showed four main clusters around “case number” (21 occurrences), “complications” (n = 13), “etiology” (n = 9) and “spread of infection” (n = 8) (Figure 3). In terms of total link strength, “etiology” and “spread of infection” were the most connected during this pandemic period (Supplementary Table S4). This likely highlights the prioritization of the scientific and medical community in understanding and controlling the pandemic. Initial discussions regarding case numbers and mortality were also more frequent during the early time within this pandemic period, but then gradually declined, possibly due to the waning of the urgency of the pandemic. In the years 1891–1894, there was a sustained interest in the clinical features and acute complications of influenza, but sub-keywords around management and therapeutics became less frequent (Supplementary Figure S1).

Close reading of the articles within this period shows that, regarding the topic of “etiology”, most of the discourse during the 1889 pandemic centred around the two dominant theories of disease transmission (miasma theory and contagion) and the underlying causative pathogen. At that time, the contagion theory was becoming more widely accepted, mostly based on observations of how the disease was spreading [36–38]. However, influenza as a causative pathogen was not yet identified and most of the authors believed that a bacterium was causing the disease, especially when various bacteria such as *Streptococcus* pyogenes and Pneumococcus were being isolated in numerous bacterial cultures or sections [39]. Influenza-related complications were also frequently discussed with a focus on pneumonia and neurological complications. For example, for the German physician Hermann Eichhorst (1849–1921) working at the University Hospital of Zurich, neurological symptoms and complications (e.g., severe fatigue, neuralgias, paralysis and body pain) were highly characteristic of the pandemic and often outlasted the respiratory symptoms [40]. Psychiatric complications were also frequently described with several case reports of patients hospitalized for symptoms (e.g., hallucinations, severe anxiety, mania and apathy) [40, 41] that some considered to be a direct sequela of influenza [41].

### 1918–1924 Pandemic Period

During the pandemic period 1918–1924 a total of 274 papers were identified, representing the highest number of articles among all observed pandemic periods. In the year 1918, 27 papers were published, followed by 79 in 1919. The highest number of articles appeared in 1920 with 70 publications, followed by 39 in 1921, 41 in 1922, 11 in 1923 and 7 in 1924. The large volume of published work, which was primarily “Research summaries” or “Original work” (Table 1), highlights the scientific interest generated by this pandemic and its considerable impact on public health, the complexity of managing it, and the urgent need to find effective therapies given the severity of the disease.

**FIGURE 4:**
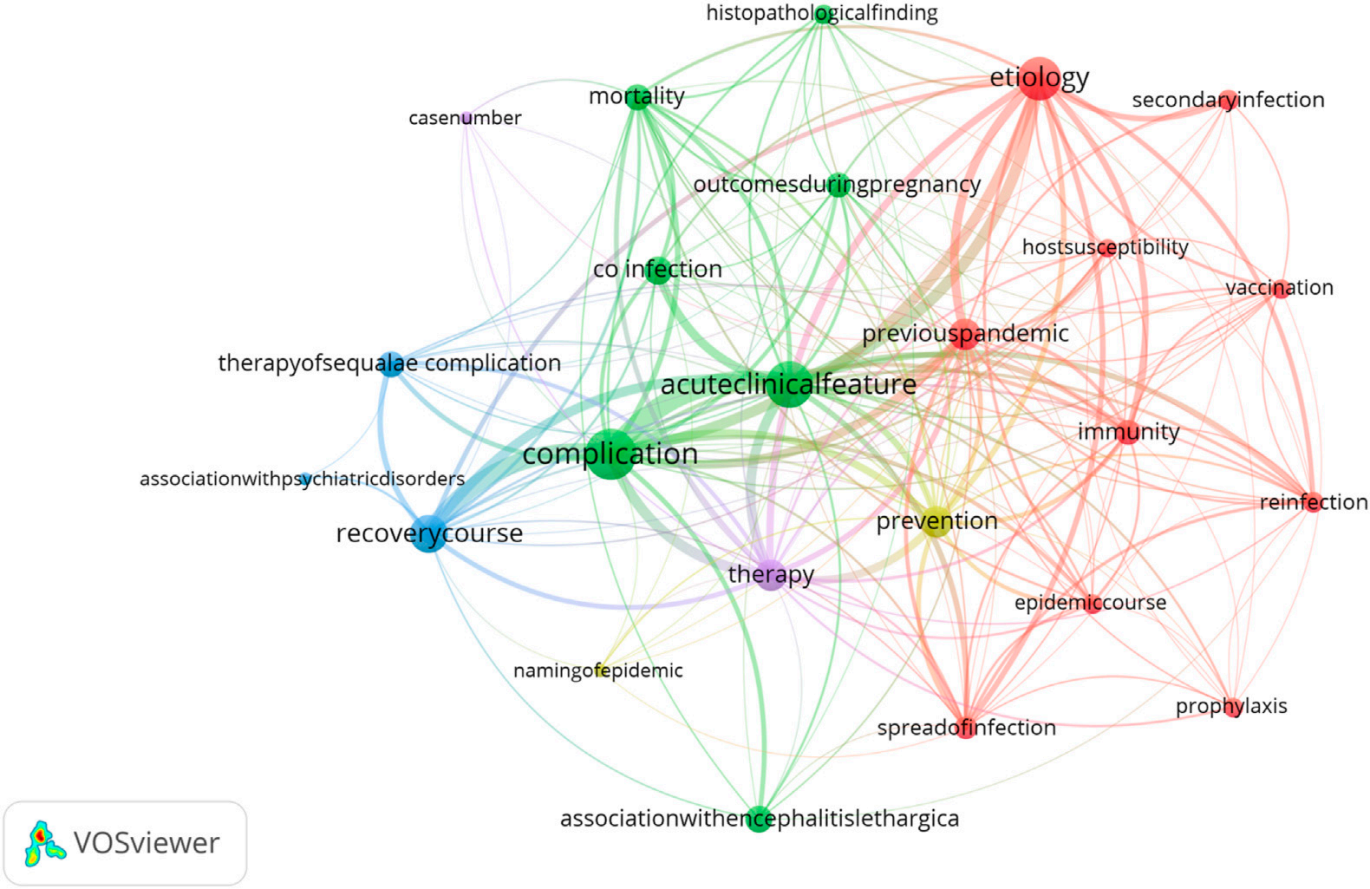
Co-occurrence and frequency map of the sub-keywords for the 1918–1924 pandemic period. The size of the dot indicates the frequency of occurrence of the given sub-keywords. Different colours refer to the different clusters identified by VOSviewer. The thickness of the connecting lines indicates the strength of co-occurrence between sub-keywords (Zurich, Switzerland. 2025).

The most prominent keyword categories during this pandemic revolved around clinical aspects, treatment, and the epidemiology of influenza (Supplementary Figure S2). The cluster analysis during this pandemic revealed three main clusters around “complication” (88 occurrences), “acute clinical feature” (n = 71), “etiology” (n = 60) and one smaller cluster around “recovery” (n = 41) (Figure 4). These sub-keywords also had the highest total link strength and were often discussed within the same context (Supplementary Table S5). Two sub-keywords that stand out during this specific pandemic period and occur less or not at all during the other periods are “association with encephalitis lethargica” and “outcomes during pregnancy”. The recurring themes in articles published during this pandemic period reflect the scientific and medical community’s struggle to understand and control the disease, the spread and severity of which were exacerbated by war and famine.

Similar to the previous pandemic, there was sustained interest among researchers in understanding the disease’s etiology and the factors driving its spread. Without knowing the causative pathogen, the widespread and diverse nature of the disease necessitated a comprehensive approach to address its challenges. At the onset of the pandemic, due to the high number of complications and deaths, there was an urgent demand for effective therapies with a surge in scientific work. Despite trying different therapies including convalescent serum [42] and some experimental treatments such as colloidal metals [43], no definitive treatment for influenza emerged and articles focused on therapies became less frequent. This emphasized the need for preventive measures such as public health strategies, good hygiene and isolation of infected individuals. During this pandemic, researchers had not yet reached a definitive conclusion about the mode of influenza transmission, but many were confident that droplet infection was the primary mode [44]. The use of masks was recommended, especially in high-risk settings such as hospitals and in crowded places [45]. However, their use was met with scepticism from others who questioned their effectiveness and practicality [46], as well as the willingness of people to comply with wearing them [47]. Public health strategies included school closures and restrictions on public gatherings. However, the necessity of such measures was debated given the economic impact of closures or gathering bans as well as the mild nature of disease in children [48, 49].

Compared to the 1889 pandemic, the 1918 pandemic was reported to be more severe with higher mortality and complications such as pneumonia complicated by secondary bacterial infections (e.g., *streptococcus* and *staphylococcus*) [50, 51]. Neurological complications, including encephalitis, meningitis, and psychosis, were frequently mentioned and sometimes persisted beyond the acute infection phase [52]. Pregnant women were particularly vulnerable with higher rates of pneumonia and other complications [53, 54], along with preterm births and increased mortality. Influenza in children was generally milder than in adults, although severe disease and complications still occurred in some [53, 55]. Encephalitis lethargica or “sleeping sickness” emerged during this period and became epidemic until late 1920s [56]. Encephalitis lethargica which primarily presented with neurological and psychiatric symptoms such as acute mental confusion, lethargy, depression and mania could last days to months and in some cases led to coma and death [57]. The relationship between Encephalitis lethargica and influenza was widely debated. Some researchers considered the two as two separate diseases [58] while others considered Encephalitis lethargica as a complication of influenza [59]. Because no such epidemic occurred after the 1920s, this question remains unanswered to this day. There were also several reports of prolonged recovery periods and post-viral symptoms such as fatigue, cough, shortness of breath [60], and hair loss [61] lasting for many weeks to months. The focus on recovery and these long-term sequalae mirrors the prolonged impact of the influenza pandemic on public health beyond the immediate acute crisis and highlights the necessity for healthcare systems to also provide adequate support for those affected by lingering post-viral symptoms and complications.

### 1957–1961 Pandemic Period

A total of 15 papers were identified between 1957 and 1961. The highest number of papers (n = 5) was published in 1958. We found three articles published during the initial stages of the pandemic in 1957 and four articles in 1959. In 1960 and 1961, we identified only one and two articles about influenza, respectively. Most of the articles (n = 9) were “Research summaries”, while four were “Original work” (Table 1). Compared to the 1918 pandemic, there were clearly fewer influenza articles, suggesting perhaps a shift in publishing practices with Swiss scientists directing influenza-related work to international or specialized journals rather than SMW, or that contextual differences (e.g., severity and fatality) between the pandemics account for the observed variation in output.

**FIGURE 5:**
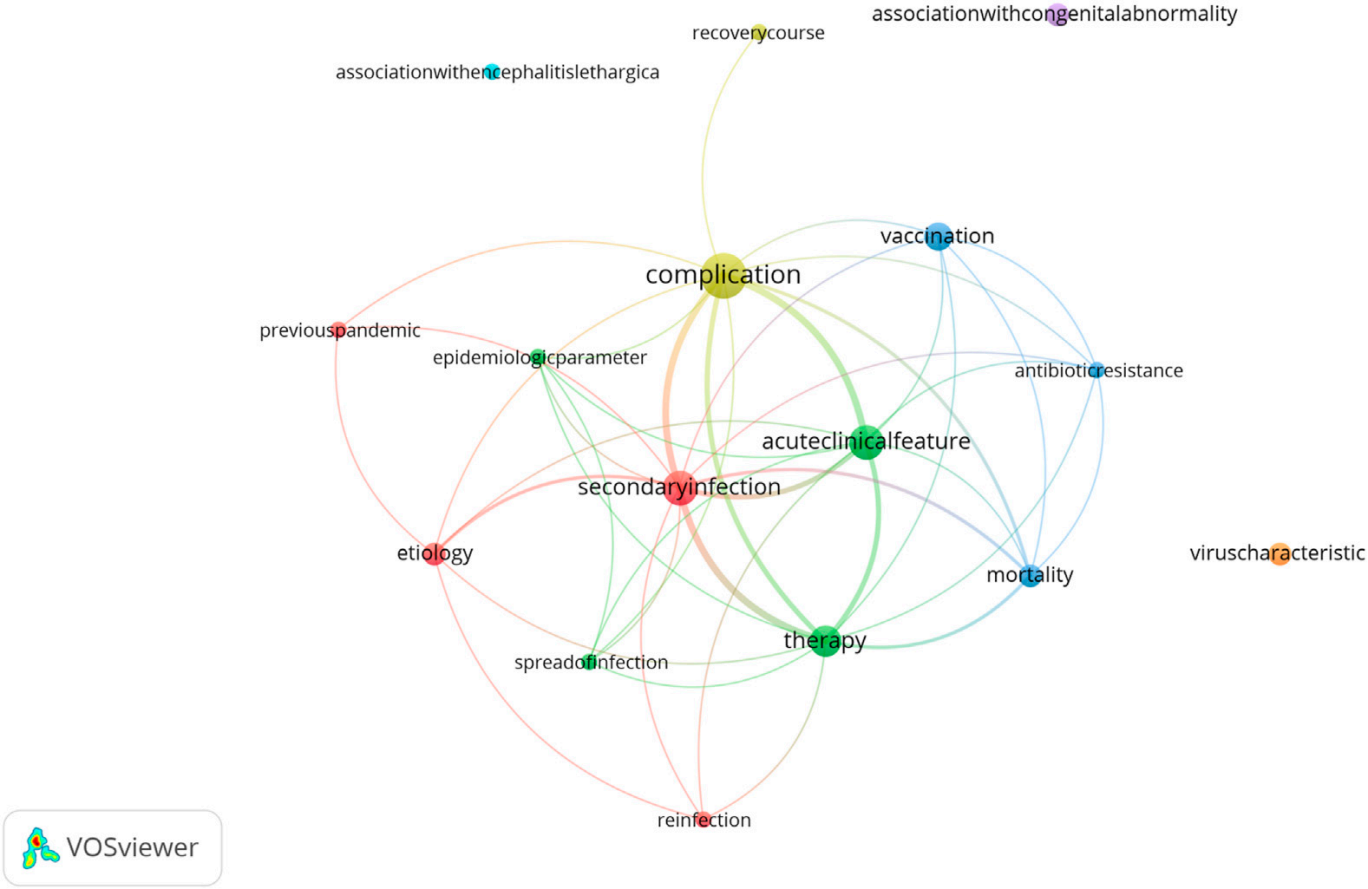
Co-occurrence and frequency map of the sub-keywords for the 1957–1961 pandemic period. The size of the dot indicates the frequency of occurrence of the given sub-keywords. Different colours refer to the different clusters identified by VOSviewer. The thickness of the connecting lines indicates the strength of co-occurrence between sub-keywords (Zurich, Switzerland. 2025).

The primary focus of the articles during this pandemic period was on management of infections and influenza-associated complications (Supplementary Figure S3), mirroring trends observed in other pandemics. In total there were 16 sub-keywords in this period. A prominent cluster was around “complication” (9 occurrences), and three other clusters were around “acute clinical feature” and “secondary infection” (n = 5 each) and “therapy” (n = 4) (Figure 5; Supplementary Table S6). “Secondary infection” had the strongest connection with “complication” and “acute clinical feature”, as indicated by the thickness of the connecting line.

Close reading of the articles from this period reveals that, although the most frequent keyword during this pandemic period is “complication”, most of the influenza infections seem to have passed without any major complications, in contrast to prior pandemics. Only a few severe cases, mostly involving pulmonary and neurological complications, were reported [62, 63]. Even though the issue of vaccination did not play a large role in SWM during this pandemic period, there were some reports emphasizing the first use of influenza vaccination during this pandemic. These reports highlighted the importance of vaccinating nursing staff, particularly vulnerable individuals, and people who live or work in large groups, such as soldiers [63, 64].

### 1968–1974 Pandemic Period

During the pandemic years of 1968–1974, a total of 12 influenza papers were found. The peak of publications for the 1968–1974 period was in 1970 with six articles. At the beginning there was only one article published in 1968 and none in 1969. After 1970, there was a decrease in the number of published articles: one article in 1971, two in 1972 and 1973 and none in 1974. Articles were mostly “Original work” followed by the “Special article” series (Table 1).

The most frequent discussed topic during this pandemic period was immunity and vaccination (Supplementary Figure S4). Close reading of the articles revealed numerous reports about the use and effectiveness of the influenza vaccination in the Swiss population. First reports on vaccination effectiveness emerged from a voluntary vaccination campaign 1970 by the national railway and postal services motivated by influenza-related staff absences during the 1969 pandemic wave [65]. The observed effectiveness in reducing infection rates and subsequently staff absences led to a vaccination campaign of all Swiss federal employees in 1971 [66]. In addition, observations suggested enhanced immunity with prior vaccination. Due to these observed medical and economic benefits, scientists recommended extending the use of vaccines to the whole population [66]. In the second half of 1972, vaccination was again offered to Swiss federal employees but a decrease in willingness to be vaccinated was noted. Reports also showed that although the vaccine did not include the new virus strain isolated in England (only the original Hongkong strain was), it still showed a benefit regarding infection susceptibility, though not for illness duration [67]. Together, these findings highlighted challenges in maintaining vaccine uptake within a population and addressing vaccine strain coverage as well as the complexity of evaluating vaccine effectiveness.

### 1977–1981 Pandemic Period

A total of seven papers were found between 1977 and 1981. Similar to the 1918, 1957 and 1968 pandemics the majority of these papers were not published in the first year of the pandemic, but rather in the second or third year. This likely reflects the typical course of pandemics which often begin in the winter of the first year and it takes some time for the scientific community to start publishing papers. The peak of publication during the 1977 influenza pandemic period was in 1978 with three papers. Most articles were “Original work” (Table 1).

During this period, as in the 1968–1974 pandemic, the most frequently discussed topic was immunity and vaccination (Supplementary Figure S5), with several studies evaluating immunity post-vaccination [68]. The discourse specifically focused on the effectiveness of vaccines in light of antigenic shifts and mutations and attribution of Guillan Barre syndrome cases and some deaths to influenza vaccination by some. Some scientists raised the question whether there was even a need for vaccines given the decrease in influenza-related deaths and whether this decrease is indeed related to vaccination or not, while others highlighted that there were still numerous deaths caused by influenza despite having a vaccine [69, 70], attributing these deaths both to the antigenic changes of the virus and the weak vaccination strategy in Switzerland [70]. This discourse mirrors similar discussions from previous and the recent COVID-19 pandemic, where similar debates arose regarding vaccine efficacy, viral mutations and the balance between vaccine related risks and benefits.

## Discussion

The evolution of scientific discourse related to influenza in SMW reflects both scientific and public health priorities and the broader sociopolitical contexts of each pandemic. Overall, there is a notable correlation between the number of articles on influenza and the number of influenza deaths in Switzerland. Scientific attention was particularly high during and after the 1889 and 1918 pandemics, but then gradually declined. However, there were also two smaller later peaks of articles and attention, one when the influenza virus was isolated and the other when vaccination and immunization in general was under discussion. Across all observed pandemics, the majority of articles were published in the second or third year. This pattern might reflect the epidemiological progression of pandemics, which usually begin in the fall/winter of the first year, with a delay before the scientific community starts publishing research.

In the 1889 and 1918 pandemics, surveillance and weekly reports dominated, aiding disease tracking and public health response. In later pandemics, original research gained prominence, reflecting a shift toward advancing scientific knowledge and shaping modern publishing. Across pandemics, “Epidemiology and disease dynamics” was the most frequent keyword category, followed by “Complications” and “Acute clinical manifestations & management.” Pneumonia and secondary infections were common concerns, especially in 1918, where such complications significantly contributed to mortality.

During the 1889 pandemic, the main focus was on disease etiology, as the causative pathogen was unknown and transmission unclear, hindering containment and public health measures [71]. The pandemic was partly a consequence of rapid globalization with railways and transoceanic ships being perfect conduits for the disease [72]. At the time, Switzerland was still a young nation, rapidly industrializing but still not very urbanized. The decentralized healthcare system meant that measures varied across cantons and the Swiss federal government’s role was modest with limited power to coordinate measures. This period also coincided with the invention and use of telegraphs and mass media [73, 74], making it the first pandemic to unfold in “real time”. Reports of outbreaks from other cities helped public health authorities and physicians to prepare for the disease’s arrival, yet this new way of communication also magnified uncertainty where reports were sometimes inaccurate, exaggerated or speculative particularly regarding causation and mortality, potentially influencing levels of trust and compliance with public health measures. This somewhat foreshadowed what we saw with COVID-19: social media was an effective tool to communicate health information to the public but also contributed to rapid and widespread dissemination of misinformation and conspiracy theories [75]. In 1918, discourse shifted to epidemiology, complications, and later, recovery. This progression reflects the scientific community’s evolving priorities—from controlling spread and complications to understanding long-term impacts. The large number of publications during this pandemic reflects also Switzerland’s neutral position during World War I. While the wartime context caused governments in many countries such as the UK, USA and Italy to restrict coverage of “bad news” or downplay severity to avoid panic and maintain morale, the pandemic was openly reported in Switzerland. Public health measures like masks, bans of mass gatherings and school closures gained importance in 1918. However, some of the measures were controversial, and their effectiveness was questioned by some professionals. Again, Switzerland’s uncoordinated and decentralized approach to the pandemic led to inconsistent approaches and sometimes to undermined containment [27, 28]. Public scepticism toward public health interventions also grew, and the economic implications became a key part of the discussion. During the COVID-19 pandemic, similar dynamics were observed where science intersected with public policy, economic pressures and varying levels of public willingness to comply with measures. The 1957 pandemic was the first pandemic to occur after the World Health Organization and the Global Influenza Surveillance Network were established [76–78]. In contrast to the previous pandemic, the 1957 pandemic saw far fewer publications. This likely reflects two shifts: first with the globalization of science after the Second World War, Swiss researchers likely increasingly published in English-language international or more specialized journals (e.g., virology or epidemiology-focused); and second, while the 1957 pandemic was also serious, influenza was by then better understood and perceived as more manageable than in 1918, which reduced the extent and scope of discussion in general medical journals. Consequently, SMW may have no longer captured the entire breadth of influenza research but instead focused on nationally relevant topics. Complications and clinical features remained key themes while etiology and transmission–key themes in 1889 and 1918 pandemics–were no longer major discussion points after the virus was isolated in the 1930s. This is also evident during the 1968 pandemic which saw limited literature focused mainly on immunization following the introduction of the first vaccines in the 1940s and broader vaccination efforts; and in the 1977 pandemic where discourse largely shifted to issues around immunity and vaccination, including vaccine efficacy, concerns regarding side effects, and implementation strategies, coinciding with the early rise of anti-vaccine movements worldwide. As understanding of viral antigens improved, vaccine formulation became a central topic, marking a major technological shift in influenza prevention.

As no comparable study exists to our knowledge, we can scarcely compare our results with others. Our study has several limitations: First, the potential for retrospective diagnosis, where we interpret past medical understanding through today’s scientific lens [79, 80]. For instance, articles were tagged using current terminology and grouped into broad modern categories. This subjectivity is both deliberate and unavoidable; we have made our method transparent so future researchers can adjust it from their own standpoint. Second, we examined only the discourse within one journal, not the broader scientific discourse. Thus, our findings may reflect the journal’s publication choices rather than general trends, especially given the increasing specialization of scientific publishing. Third, our analysis combines bibliometric visualization with close reading. Future steps should include digitizing the journal’s full archive to allow access to complete texts. This would enable distant reading, where shifts in discourse could be more objectively traced using text mining, topic modelling, and related methods. Due to the scope of the study, it was not possible to conduct a systematic qualitative discourse analysis of the medical literature beyond SMW and to integrate our bibliometric findings into this discourse. For the study to go beyond its descriptive nature and become more interpretative, this important contextualization must be addressed in a follow-up project. Finally, given the continuities we observed across past pandemic periods, comparing them with more recent events like the 2009 H1N1 influenza or the COVID-19 pandemic would be worthwhile.

This study is a first attempt to trace such influenza-related scientific discourse changes in one journal and topic, and broader generalisation requires comparative analysis of other journals and topics. Nevertheless, we show that certain continuities emerged during and after pandemics, despite major medical advances and changing living conditions. Scientific uncertainty as well as debates around public health measures and vaccination have not been unique to COVID-19 but rather recurring themes for more than a century across several pandemics. Recognizing this is not only important for understanding past responses but also helps to anticipate future challenges and supports preparedness for emerging health threats.
