# Supplementary material for: Reconstruction of the Discourse on Influenza During Pandemics Between 1889 and 1980 in the Predecessor Journal of Swiss Medical Weekly - A Narrative Review and Bibliometric Analysis
Source: Public Health Rev. 2025 Oct 21;46:1608522. doi: 10.3389/phrs.2025.1608522 (PMC12583112; doi:10.3389/phrs.2025.1608522)
Supplement: Supplementary file 2 [file Supplementaryfile1.docx]

**Supplementary Material**

**Reconstruction of the discourse on influenza during pandemics between 1889 and 1980 in the predecessor journal of Swiss Medical Weekly - a narrative review and bibliometric analysis**

**The journal selected as source of information**

Today's SMW is the most important medical journal in Switzerland. The journal was and still is an important part of Swiss scientific history. According to the editorial of the very first issue of the Correspondenzblatt der Schweizer Ärzte in January 1871, the journal set itself the task of fostering dialogue between exponents of medical knowledge and practice. The explicit intention was to address general social and scientific issues affecting the medical profession and Swiss physicians in particular, and to publish original reports from practice and hospitals, information from the authorities and associations, papers and reviews of medical work, reports on local or cantonal medical conditions and initially reports on the occurrence of epidemic and endemic diseases.[1] From the 1890s onwards with the emergence of weekly official statistical publications, the journal no longer published reports on the occurrence of epidemic and endemic diseases. In 1920, the journal was renamed “Schweizerische medizinische Wochenschrift” (SMWo) and political issues relating to the standing of the medical profession were from then on addressed in a separate professional bulletin.[2] However, SMWo itself continued to publish original and other scientific articles as before and the publication frequency increased over time. Until 1909, 24 issues of the journal were published per year, from 1910 to 1912 36 issues per year, and finally, from 1913 it became a weekly publication with 52 issues per year. In 2001, the SMWo was split into the “Swiss Medical Forum”, an educational journal for doctors, and SMW.[2] While full texts from the late 1990s onwards are now publicly accessible on the SMW website, earlier issues and articles have not yet been digitised from the paper volumes archived in libraries.[3]

**Summary of the selected pandemic periods from a Swiss perspective**

**1889-1894**

The 1889 flu pandemic, also commonly referred to as the “Russian flu”, is considered the first influenza pandemic that was documented to be global [4]. It likely originated in Russia and from there spread westwards across Europe and later the whole world, causing over 1 million deaths [5,6]. This pandemic was marked by a high morbidity, but a relatively low rate of mortality [7,8]. In Switzerland, excess mortality was observed during the winters of 1889-1890 and 1890-1891 among individuals over the age of 70, while no excess mortality was noted in younger age groups [9,10]. During this time, several waves of influenza occurred in Switzerland, namely in the winter months of 1889/1890 and 1893/1894 [11]. The pandemic took place during a period of expansion of the railway network which facilitated the spread of the virus across the country and worldwide [8]. Little is known about the causative pathogen, as there are very few human remain samples from this period, but an influenza A virus (H2N2 or H3N8) was long assumed to be the causative pathogen [12]. During the COVID-19 pandemic the question emerged as to whether the pandemic was caused by a coronavirus rather than an influenza virus [13]. This question will remain open until genetic evidence is available.

**1918-1924**

The 1918 influenza pandemic, often referred to as the “Spanish flu”, is considered one of the most devastating pandemics in history, leading to an estimated 100 million deaths worldwide [14]. Although the name would suggest that the pandemic originated in Spain, its origins are still the subject of ongoing research, and many believe that the virus originated in the United States and spread to Europe with soldiers traveling to the western Front during World War I [7,15]. The pandemic unfolded in three waves: the first wave appeared in summer of 1918 and infected a large portion of the population with relatively few deaths [16]. However, during the summer of 1918, the virus mutated, leading to a much more lethal second wave from October to December 1918, which accounted for two-thirds of the total deaths. The third wave took place in the winter of 1918-1919, and there was a strong later wave in February 1920 [7]. In Switzerland, three distinct waves were identified: in July 1918, in October to December 1918 and in February 1920 [17] with a total of ca. 25000 deaths [18]. In contrast to other pandemics where there is a high mortality rate in children and the elderly, the peak of excess deaths during this pandemic occurred uncharacteristically among young adults between 20 and 40 years of age, the reasons of which are still not clear [9,19,20]. Women, especially pregnant women, were particularly vulnerable to influenza and faced a higher risk of mortality [7].

**1957-1961**

The “Asian Flu” was a global influenza pandemic that emerged in 1957 and was caused by the H2N2 influenza virus [6]. The novel flu strain, which originated from an avian influenza virus, was first identified in the Yunnan Province in China in February 1957 [6,21]. The virus first spread in East Asia before quickly spreading worldwide during the summer. Its rapid dissemination was facilitated primarily by land and sea routes, while air travel played a negligible role in its transmission [21]. The second wave was much stronger than the first, a phenomenon that could be observed in the 1918 influenza pandemic, but also during the COVID-19 pandemic [21,22]. Compared to the 1918 pandemic, the 1957 pandemic was less severe in terms of mortality, but it still resulted in approximately 1.1. million deaths worldwide, with some estimates as high as 2 million deaths [23,24]. Younger age groups were more affected than older ones, indicating that older individuals had some pre-existing immunity [21].

The 1957 pandemic was the first to be monitored and observed on a global scale, thanks to the establishment of worldwide centers dedicated to tracking the virus. Additionally, a global network of laboratories was integrated into the Influenza Research Center in London in 1957, enabling the rapid study of the virus strain [21]. This was also the first instance in which the effects of an influenza vaccine could be recorded in certain subgroups of the population [25]. Even though the vaccination efficacy was 53-60%, the vaccination could not have a significant influence on the course of the pandemic, as the coverage of the population with the vaccination was insufficient and the vaccination came too late in Europe [21].

**1968-1974**

Just ten years after the “Asian flu”, an antigenic shift in the “Asian influenza” strain led to the “Hong Kong influenza pandemic”, with the A/H3N2 virus first being isolated in Hong Kong in July 1968 [21,26]. The spread of the virus was partly facilitated by Vietnam War veterans returning to the U.S [21]. Unlike previous pandemics, this pandemic was significantly propagated through international air travel [26]. The virus’s most characteristic feature was its high transmissibility, although it was milder than the Asian flu virus [21]. In Canada and the United States, there was a large wave in 1968-69, while the second wave in 1969/70 was milder. In contrast, Europe, Australia and Asia experienced higher mortality rates in the second wave than in the first [26]. Globally, the pandemic resulted in an estimated 500’000 to 2 million deaths, with a higher mortality rate observed among younger people, particularly in young children [21]. In Switzerland, morbidity and mortality in these two waves were not significantly higher as during stronger seasonal influenza waves in the years before [18].

**1977-1981**

The reemergence of the H1N1 virus in 1977, known as the “Russian flu”, was first reported by the Soviet Union to the World Health Organization (WHO) [27]. While the Soviet Union was the first to notify the WHO of the outbreak, the virus strain had been isolated in three different locations in China as early as May 1977, from where it quickly spread worldwide [27,28]. The disease was notably mild, with no significant increase in mortality; in fact, the mortality rate was even lower than that of a typical seasonal influenza, preventing the outbreak from escalating into a full-blown pandemic [21,28]. Notably, individuals under the age of 26 were more susceptible to the virus, likely due to a lack of prior immunity, unlike the older generation. The origin of the virus remains controversial, with some suggesting a natural reemergence while others propose theories including a deliberate release, a failed vaccine trial, or a laboratory accident, especially considering the virus's similarity to a 1950 strain and its mild clinical symptoms. The theory of a deliberate release of the virus lies in the context of the Cold War, when the Soviet Unition devoted many resources to the development of biological weapons and the virus’s impact on young adults, a key demographic in the military, raises suspicions. Another theory concerns that the virus may have originated from vaccination trials involving attenuated viruses, where improper attenuation could have triggered a global outbreak. However, the most widely accepted theory is that a laboratory accident resulted in a biosafety incident, leading to the virus’s reemergence [28].

Bibliography

1. Klinische Forschung in der Schweiz : 125 Jahre Schweizerische Medizinische Wochenschrift. 125 Jahre Schweizerische Medizinische Wochenschrift. Basel: Schwabe; 1995.

2. Wolff E. Vom Kampfblatt zum Flaggschiff. Schweizerische Ärztezeitung. 2020 Jan 8;

3. Aguzzi A, Waeber G. Swiss Medical Weekly: Quo vadis? Swiss Med Wkly. 2022 Nov 7;152(4546):40030.

4. Kousoulis AA. The 1889-90 flu pandemic in Greece: a social, cultural and economic history with lessons for the 21st century. Infez Med. 2023;31(3):411–20.

5. Erkoreka A, Hernando-Pérez J, Ayllon J. Coronavirus as the Possible Causative Agent of the 1889–1894 Pandemic. Infect Dis Rep. 2022 Jun 13;14(3):453–69.

6. Noor R, Maniha SM. A brief outline of respiratory viral disease outbreaks: 1889–till date on the public health perspectives. Virusdisease. 2020 Dec 2;31(4):441–9.

7. Mamelund SE. Influenza, Historical. In: International Encyclopedia of Public Health. Elsevier; 2008. p. 597–609.

8. Goff JM Le. Diffusion of influenza during the winter of 1889-1890 in Switzerland. Genus [Internet]. 2011;67(2):77–99. Available from: http://www.jstor.org/stable/genus.67.2.77

9. Matthes KL, Floris J, Merzouki A, Junker C, Weitkunat R, Rühli F, et al. Spatial pattern of all cause excess mortality in Swiss districts during the pandemic years 1890, 1918 and 2020. medRxiv [Internet]. 2024 Jan 1;2024.02.13.24302701. Available from: http://medrxiv.org/content/early/2024/02/13/2024.02.13.24302701.abstract

10. Suter J, Devos I, Matthes K, Staub K. The health and demographic impacts of the “Russian flu” pandemic in Switzerland in 1889/1890 and in the years thereafter. Epidemiol Infect.

11. Schmid Francke. Die Influenza in der Schweiz in den Jahren 1889-1894 : auf Grund amtlicher Berichte und sonstigen Materials / dargestellt von F. Schmid. Bern: Stämpfli; 1895.

12. Gagnon A, Acosta JE, Madrenas J, Miller MS. Is Antigenic Sin Always “Original?” Re-examining the Evidence Regarding Circulation of a Human H1 Influenza Virus Immediately Prior to the 1918 Spanish Flu. PLoS Pathog. 2015 Mar 5;11(3):e1004615.

13. Berche P. The enigma of the 1889 Russian flu pandemic: A coronavirus? Presse Med. 2022 Sep;51(3):104111.

14. Johnson NPAS, Mueller J. Updating the Accounts: Global Mortality of the 1918-1920 “Spanish” Influenza Pandemic. Bull Hist Med. 2002 Mar;76(1):105–15.

15. Barry JM. The site of origin of the 1918 influenza pandemic and its public health implications. J Transl Med. 2004;2(1):3.

16. Humphries MO. Paths of Infection: The First World War and the Origins of the 1918 Influenza Pandemic. War Hist. 2014 Jan 8;21(1):55–81.

17. Zürcher K, Zwahlen M, Ballif M, Rieder HL, Egger M, Fenner L. Influenza Pandemics and Tuberculosis Mortality in 1889 and 1918: Analysis of Historical Data from Switzerland. PLoS One. 2016 Oct 5;11(10):e0162575.

18. Staub K, Ruhli F, Floris J. The “Pandemic Gap” in Switzerland across the 20th century and the necessity of increased science communication of past pandemic experiences. Swiss Med Wkly. 2020 Jan 1;

19. Taubenberger JK, Morens DM. 1918 Influenza: the Mother of All Pandemics. Emerg Infect Dis. 2006 Jan;12(1):15–22.

20. Morens DM, Taubenberger JK, Harvey HA, Memoli MJ. The 1918 influenza pandemic: Lessons for 2009 and the future. Crit Care Med. 2010 Apr;38:e10–20.

21. Saunders-Hastings P, Krewski D. Reviewing the History of Pandemic Influenza: Understanding Patterns of Emergence and Transmission. Pathogens. 2016 Dec 6;5(4):66.

22. Vilella A, Trilla A. The COVID-19 Pandemic—an Epidemiological Perspective. Curr Allergy Asthma Rep. 2021 Apr 28;21(4):29.

23. Guan Y, Vijaykrishna D, Bahl J, Zhu H, Wang J, Smith GJD. The emergence of pandemic influenza viruses. Protein Cell. 2010 Jan 7;1(1):9–13.

24. Viboud C, Simonsen L, Fuentes R, Flores J, Miller MA, Chowell G. Global Mortality Impact of the 1957–1959 Influenza Pandemic. Journal of Infectious Diseases. 2016 Mar 1;213(5):738–45.

25. Kilbourne ED. Influenza Pandemics of the 20th Century. Emerg Infect Dis. 2006 Jan;12(1):9–14.

26. Viboud C, Grais RF, Lafont BAP, Miller MA, Simonsen L. Multinational Impact of the 1968 Hong Kong Influenza Pandemic: Evidence for a Smoldering Pandemic. J Infect Dis. 2005 Jul 15;192(2):233–48.

27. Kalyar F, Chen X, Chughtai AA, MacIntyre CR. Origin of the H1N1 (Russian influenza) pandemic of 1977—A risk assessment using the modified Grunow–Finke tool (mGFT). Risk Analysis. 2024 Jun 9;

28. Rozo M, Gronvall GK. The Reemergent 1977 H1N1 Strain and the Gain-of-Function Debate. mBio. 2015 Sep;6(4).

**Supplementary Figure S1:** Relative proportion of the five broad keyword categories per year with 100% representing the total mentions for the whole 1889-1894 pandemic period. (Zurich, Switzerland. 2025)


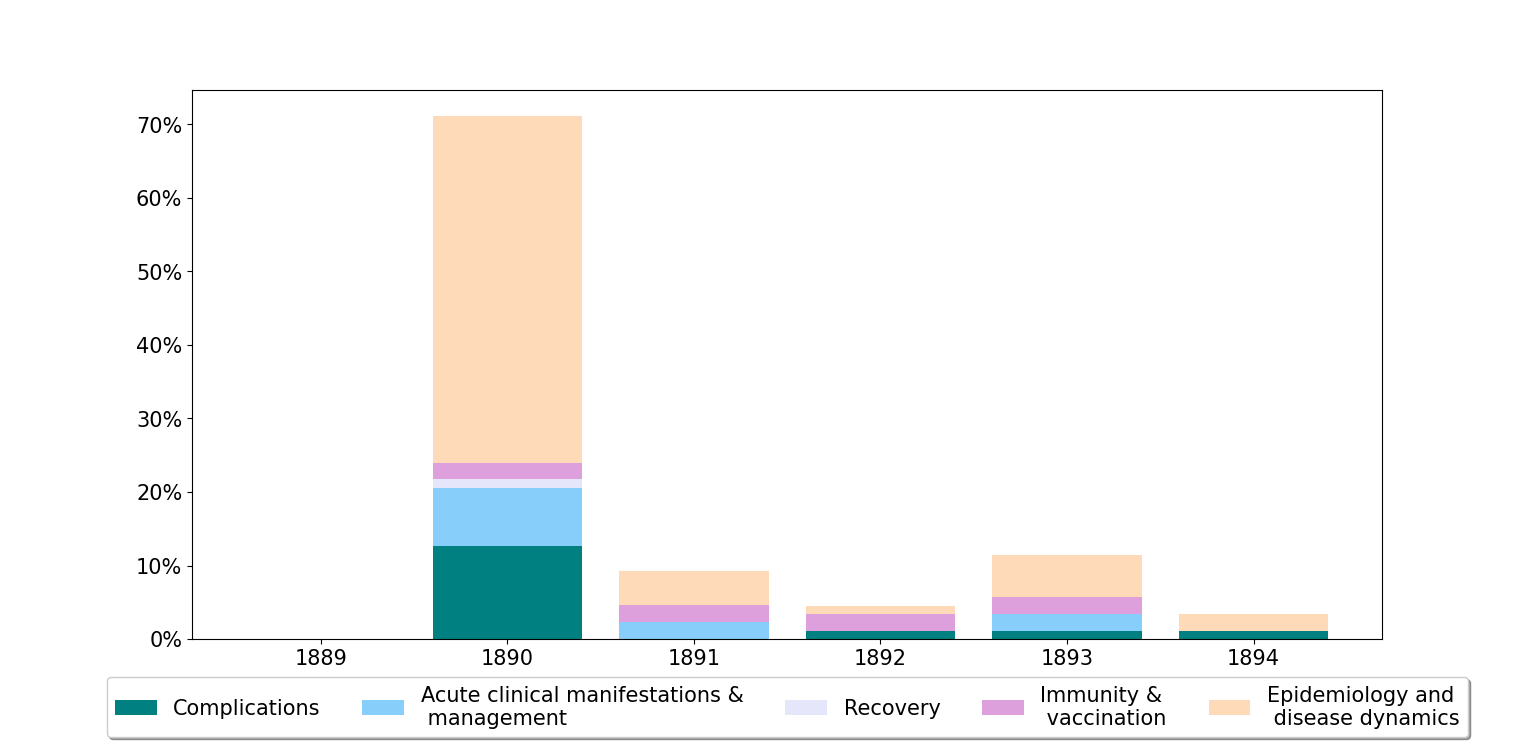


**Supplementary Figure S2:** Relative proportion of the five broad keyword categories per year with 100% representing the total mentions for the whole 1918-1924 pandemic period. (Zurich, Switzerland. 2025)


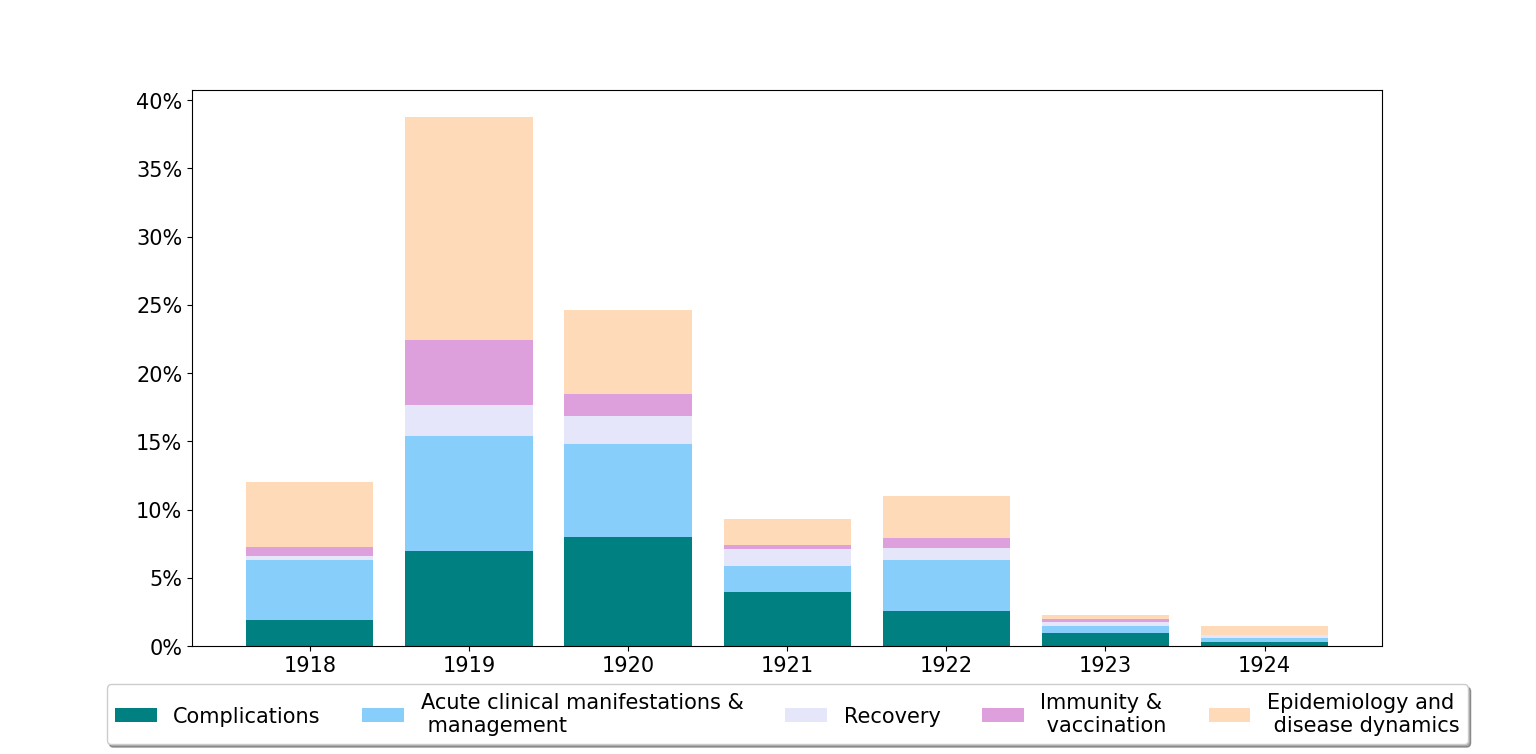


**Supplementary Figure S3:** Relative proportion of the five broad keyword categories per year with 100% representing the total mentions for the whole 1957-1961 pandemic period. (Zurich, Switzerland. 2025)


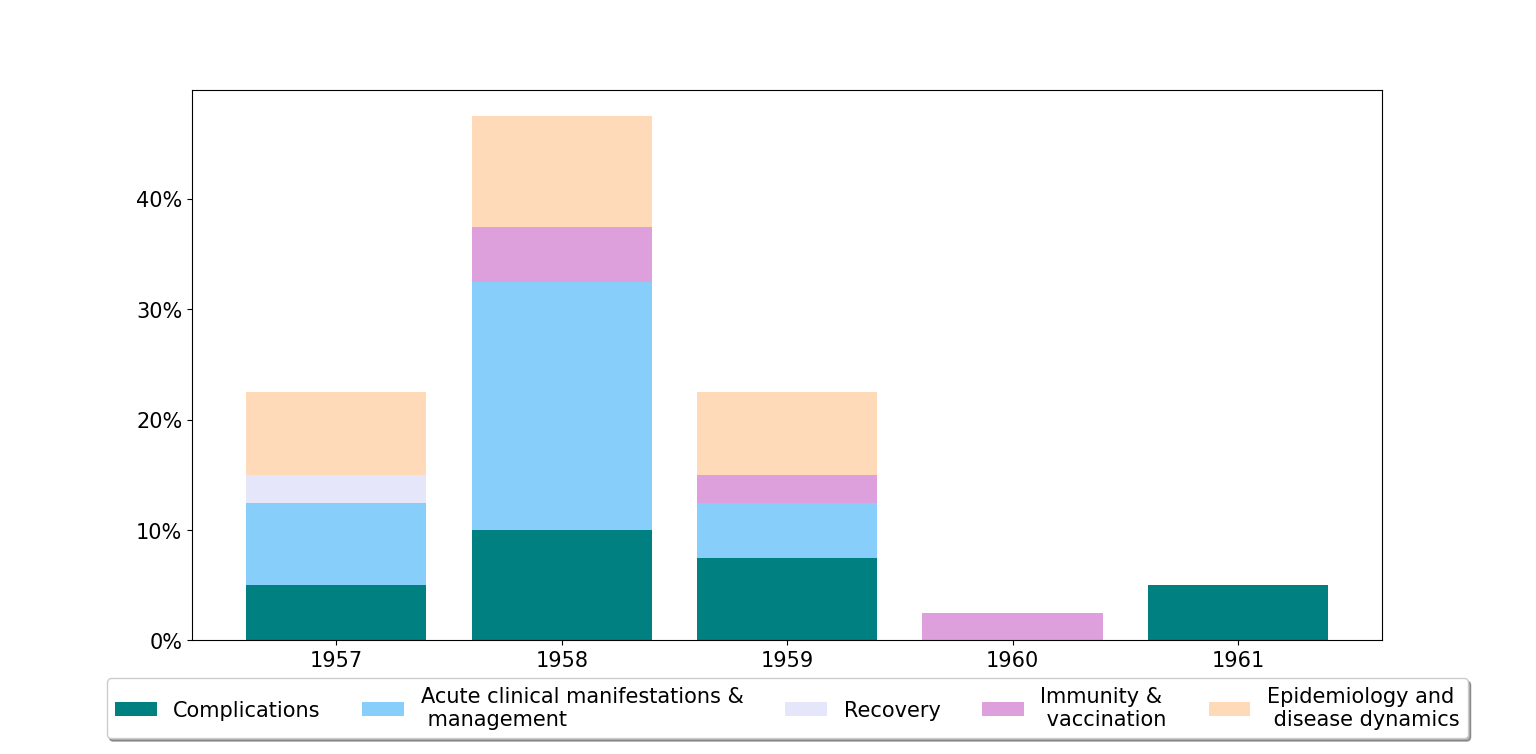


**Supplementary Figure S4:** Relative proportion of the five broad keyword categories per year with 100% representing the total mentions for the whole 1968-1974 pandemic period. (Zurich, Switzerland. 2025)


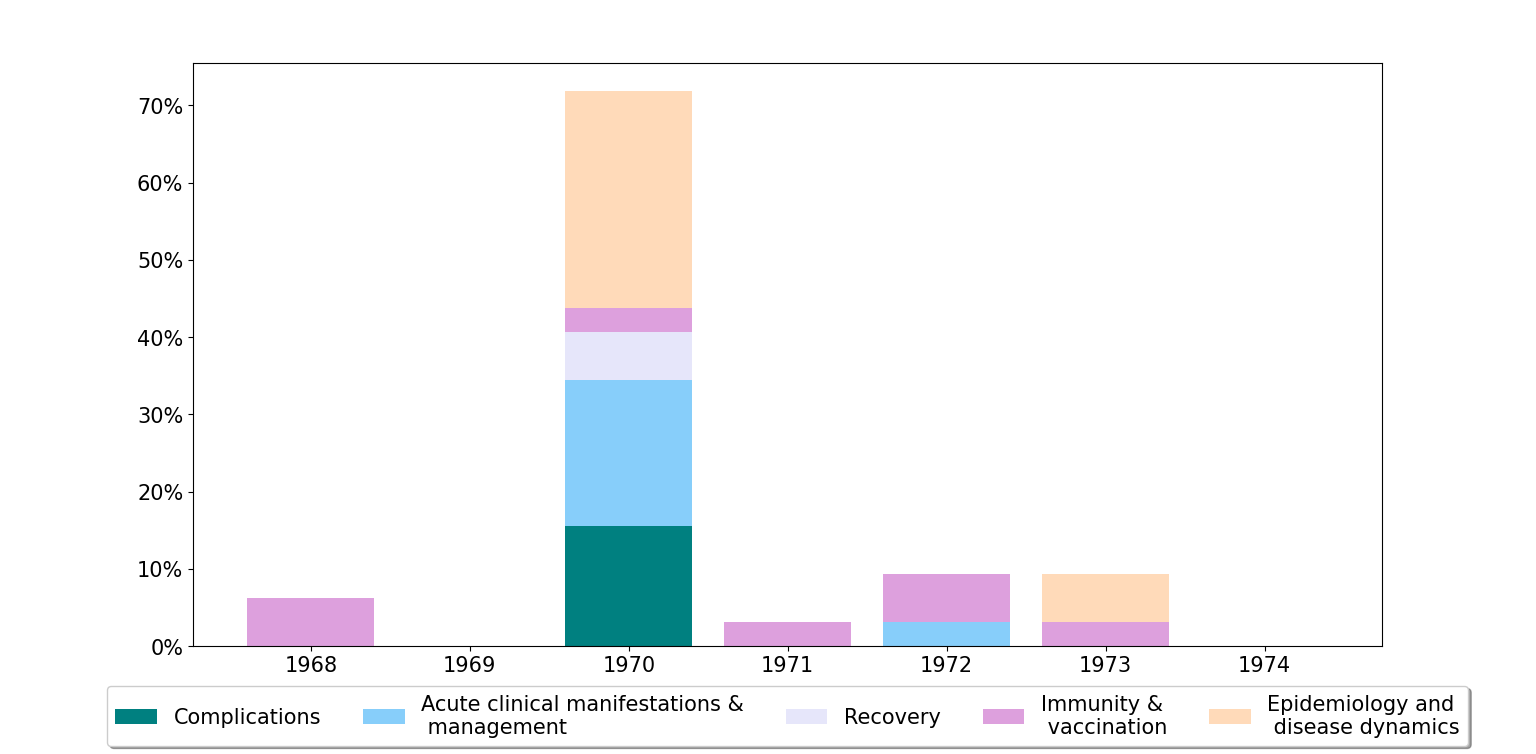


**Supplementary Figure S1:** Relative proportion of the five broad keyword categories per year with 100% representing the total mentions for the whole 1977-1981 pandemic period. (Zurich, Switzerland. 2025)


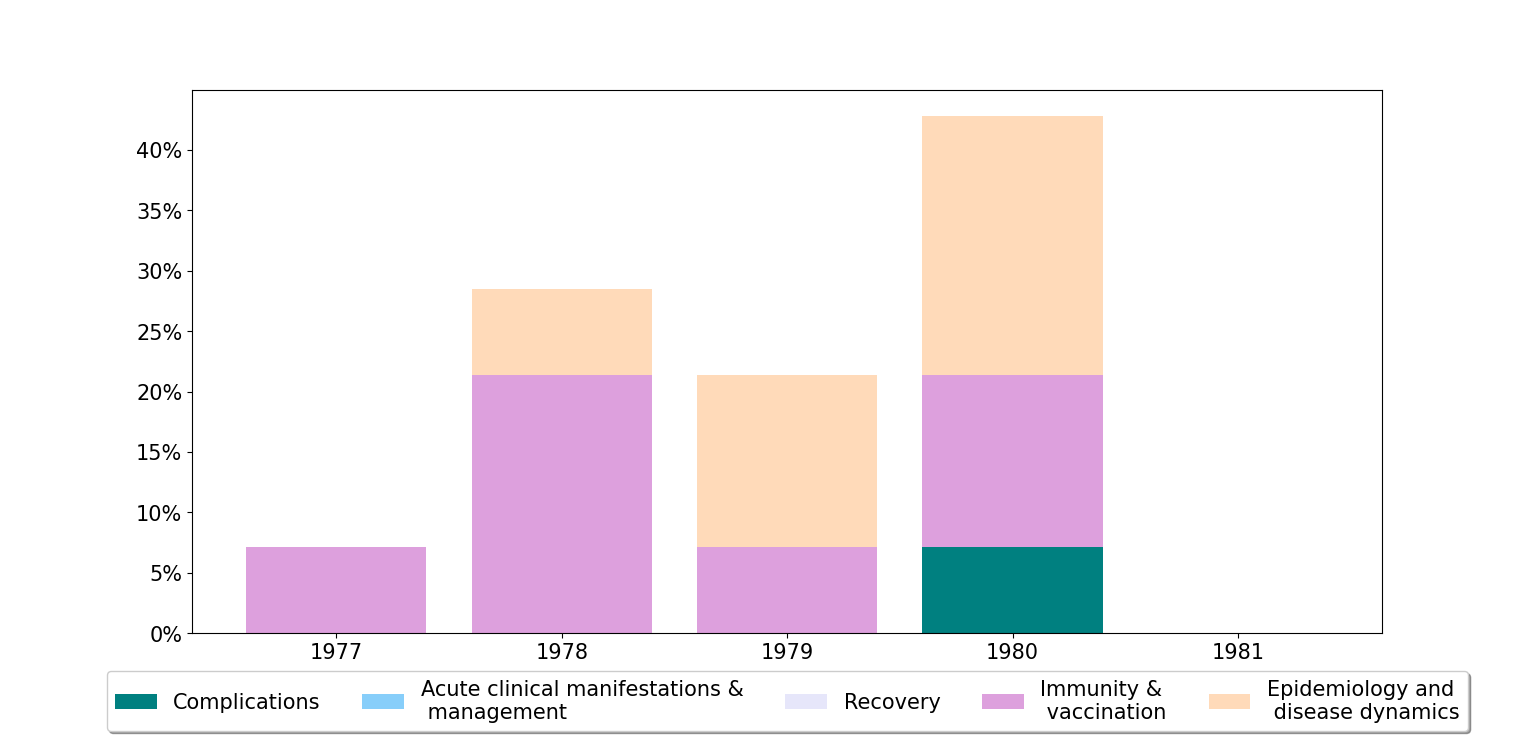


*(Supplementary Table S1 und Supplementary Table S3 are uploaded as separate excel files).*

**Supplementary Table S2**: The 31 sub-keywords and their categorization into the broad categories. (Zurich, Switzerland. 2025)

| **Broad category** | **Sub-keyword** |
| --- | --- |
| Acute clinical manifestations & management | Acute clinical feature |
|  | Co-infection |
|  | Secondary infection |
|  | Therapy |
| Complications | Association with congenital abnormalities |
|  | Association with Encephalitis lethargica |
|  | Association with psychiatric disorders |
|  | Complication |
|  | Outcomes during pregnancy |
|  | Therapy of sequalae/Complication |
| Epidemiology and disease dynamics | Antibiotic resistance |
|  | Case number |
|  | Epidemic course |
|  | Epidemiologic parameter |
|  | Etiology |
|  | Histopathological finding |
|  | Influenza-like illness |
|  | Mortality |
|  | Naming of epidemic |
|  | Prevention |
|  | Previous pandemic |
|  | Rumors on nature of disease |
|  | Spread of infection |
|  | Strain on healthcare system |
|  | Virus characteristic |
| Immunity & vaccination | Host susceptibility |
|  | Immunity |
|  | Prophylaxis |
|  | Reinfection |
|  | Vaccination |
| Recovery | Recovery course |

**Appendix Table S4**: The different clusters with the occurrence of the sub-keywords, the number of links and the total link strength for the 1889-1894 pandemic period taken from the VOSviewer analysis. (Zurich, Switzerland. 2025)

| **Cluster 1** | **Sub-keyword** | **Occurrence** | **Number of links** | **Total Link Strength** |
| --- | --- | --- | --- | --- |
|  | Etiology | 9 | 13 | 30 |
|  | Spread of infection | 8 | 12 | 25 |
|  | Recovery course | 1 | 4 | 4 |
|  | Epidemic course | 1 | 3 | 3 |
|  |  |  |  |  |
|  |  |  |  |  |
| **Cluster 2** | **Sub-keyword** | **Occurrence** | **Number of links** | **Total Link Strength** |
|  | Complication | 13 | 12 | 26 |
|  | Mortality | 3 | 10 | 15 |
|  | Reinfection | 3 | 10 | 14 |
|  | Host susceptibility | 1 | 8 | 8 |
|  | Immunity | 1 | 6 | 6 |
|  |  |  |  |  |
|  |  |  |  |  |
| **Cluster 3** | **Sub-keyword** | **Occurrence** | **Number of links** | **Total Link Strength** |
|  | Prevention | 2 | 10 | 10 |
|  | Therapy | 3 | 7 | 7 |
|  | Association with psychiatric disorders | 1 | 3 | 3 |
|  | Previous pandemic | 2 | 3 | 3 |
|  |  |  |  |  |
|  |  |  |  |  |
| **Cluster 4** | **Sub-keyword** | **Occurrence** | **Number of links** | **Total Link Strength** |
|  | Acute clinical feature | 5 | 13 | 23 |
|  | Co-infection | 1 | 1 | 1 |
|  |  |  |  |  |
|  |  |  |  |  |
| **Cluster 5** | **Sub-keyword** | **Occurrence** | **Number of links** | **Total Link Strength** |
|  | Case number | 21 | 13 | 26 |
|  | Influenza-like illness | 1 | 1 | 1 |
|  |  |  |  |  |
|  |  |  |  |  |
| **Cluster 6** | **Sub-keyword** | **Occurrence** | **Number of links** | **Total Link Strength** |
|  | Epidemiological parameter | 4 | 9 | 15 |
|  |  |  |  |  |
|  |  |  |  |  |
| **Cluster 7** | **Sub-keyword** | **Occurrence** | **Number of links** | **Total Link Strength** |
|  | Prophlaxis | 2 | 0 | 0 |
|  |  |  |  |  |
|  |  |  |  |  |
| **Cluster 8** | **Sub-keyword** | **Occurrence** | **Number of links** | **Total Link Strength** |
|  | Vaccination | 1 | 0 | 0 |

**Appendix Table S5**: The different clusters with the occurrence of the sub-keywords, the number of links and the total link strength for the 1918-1914 pandemic period taken from the VOSviewer analysis. (Zurich, Switzerland. 2025)

| **Cluster 1** | **Sub-keyword** | **Occurrence** | **Number of links** | **Total Link Strength** |
| --- | --- | --- | --- | --- |
|  | Etiology | 60 | 21 | 116 |
|  | Previous pandemic | 26 | 21 | 81 |
|  | Spread of infection | 12 | 20 | 56 |
|  | Immunity | 15 | 16 | 55 |
|  | Reinfection | 9 | 18 | 46 |
|  | Epidemic course | 8 | 15 | 36 |
|  | Host susceptibility | 7 | 16 | 31 |
|  | Vaccination | 8 | 16 | 30 |
|  | Secondary infection | 9 | 10 | 19 |
|  | Prophylaxis | 8 | 12 | 18 |
|  |  |  |  |  |
|  |  |  |  |  |
| **Cluster 2** | **Sub-keyword** | **Occurrence** | **Number of links** | **Total Link Strength** |
|  | Complication | 88 | 22 | 151 |
|  | Acute clinical feature | 71 | 22 | 145 |
|  | Mortality | 16 | 19 | 60 |
|  | Co-infection | 21 | 14 | 38 |
|  | Outcomes during pregnancy | 15 | 17 | 34 |
|  | Histopathological finding | 8 | 13 | 23 |
|  | Association with Encephalitis lethargica | 18 | 10 | 20 |
|  |  |  |  |  |
|  |  |  |  |  |
| **Cluster 3** | **Sub-keyword** | **Occurrence** | **Number of links** | **Total Link Strength** |
|  | Recovery course | 41 | 19 | 81 |
|  | Therapy of sequalae/complication | 17 | 13 | 32 |
|  | Association with psychiatric disorders | 3 | 2 | 4 |
|  |  |  |  |  |
|  |  |  |  |  |
| **Cluster 4** | **Sub-keyword** | **Occurrence** | **Number of links** | **Total Link Strength** |
|  | Therapy | 26 | 22 | 99 |
|  | Case number | 3 | 6 | 8 |
|  |  |  |  |  |
|  |  |  |  |  |
| **Cluster 5** | **Sub-keyword** | **Occurrence** | **Number of links** | **Total Link Strength** |
|  | Prevention | 26 | 19 | 81 |
|  | Naming of epidemic | 3 | 9 | 10 |

**Appendix Table S6**: The different clusters with the occurrence of the sub-keywords, the number of links and the total link strength for the 1957-1961 pandemic period taken from the VOSviewer analysis. (Zurich, Switzerland. 2025)

| **Cluster 1** | **Sub-keyword** | **Occurrence** | **Number of links** | **Total Link Strength** |
| --- | --- | --- | --- | --- |
|  | Secondary infection | 5 | 11 | 21 |
|  | Etiology | 2 | 6 | 7 |
|  | Reinfection | 1 | 4 | 4 |
|  | Previous pandemic | 1 | 3 | 3 |
|  |  |  |  |  |
|  |  |  |  |  |
| **Cluster 2** | **Sub-keyword** | **Occurrence** | **Number of links** | **Total Link Strength** |
|  | Therapy | 4 | 10 | 18 |
|  | Acute clinical feature | 5 | 10 | 17 |
|  | Epidemiologic parameter | 1 | 5 | 5 |
|  | Spread of infection | 1 | 5 | 5 |
|  |  |  |  |  |
|  |  |  |  |  |
| **Cluster 3** | **Sub-keyword** | **Occurrence** | **Number of links** | **Total Link Strength** |
|  | Mortality | 2 | 6 | 9 |
|  | Antibiotic resistance | 1 | 6 | 6 |
|  | Vaccination | 3 | 6 | 6 |
|  |  |  |  |  |
|  |  |  |  |  |
| **Cluster 4** | **Sub-keyword** | **Occurrence** | **Number of links** | **Total Link Strength** |
|  | Complication | 9 | 11 | 20 |
|  | Recovery course | 1 | 1 | 1 |
|  |  |  |  |  |
|  |  |  |  |  |
| **Cluster 5** | **Sub-keyword** | **Occurrence** | **Number of links** | **Total Link Strength** |
|  | Association with congenital abnormality | 2 | 0 | 0 |
|  |  |  |  |  |
|  |  |  |  |  |
| **Cluster 6** | **Sub-keyword** | **Occurrence** | **Number of links** | **Total Link Strength** |
|  | Association with Encephalitis lethargica | 1 | 0 | 0 |
|  |  |  |  |  |
|  |  |  |  |  |
| **Cluster 7** | **Sub-keyword** | **Occurrence** | **Number of links** | **Total Link Strength** |
|  | Virus characteristic | 2 | 0 | 0 |
